# Analysis of E-cigarette use in the 2014 Eurobarometer survey: calling out deficiencies in epidemiology methods

**DOI:** 10.1007/s11739-017-1667-z

**Published:** 2017-05-05

**Authors:** Riccardo Polosa, Pasquale Caponnetto, Ray Niaura, David Abrams

**Affiliations:** 1 Centro Prevenzione e Cura del Tabagismo, Azienda Ospedaliero, Universitaria ‘‘Policlinico-V. Emanuele’’, Università di Catania, Catania, Italy; 2 Dipartimento di Medicina Clinica e Sperimentale, Università di Catania, Azienda Ospedaliero, Universitaria ‘‘Policlinico-Vittorio Emanuele’’, Università di Catania, Catania, Italy; 3 Institute for Social Marketing, University of Stirling, Stirling, UK; 4 The Schroeder Institute for Tobacco Research and Policy Studies at Truth Initiative, Washington, DC, USA; 5 Department of Health, Behavior and SocietyJohns Hopkins Bloomberg School of Public Health, Baltimore, MD USA

**Keywords:** Electronic cigarette, Smoking, Epidemiology

Smoking is a difficult addiction to break with many smokers persisting in tobacco use for numerous years, and typically cycling through multiple periods of remission and relapse [1]. Yet, while smoking cessation may be the most desirable final outcome, substitution of conventional cigarettes by alternative non-combusted forms of nicotine delivery, such as electronic cigarettes (ECs), is now a realistic compromise that is likely to eliminate or substantially reduce exposure to tobacco smoke toxicants [2].

ECs were first marketed 10 years ago, and are now widely used as long-term substitutes for tobacco cigarettes primarily because they share sufficient similarities with bio-behavioural and sensorial aspects of smoking [3]. Designed to enable inhalation of flavoured liquids, in which pharmaceutical grade nicotine may (or may not) be added, these battery-operated vaporizers do not contain tobacco. Nonetheless, ECs are now legally considered to be tobacco products in the European Union and in the United States.

The scientific evidence indicates that ECs are much less harmful than conventional cigarettes [4, 5]. Despite their reduced risk profile, questions remain as to whether ECs are effective aids for smoking cessation, promote uptake by nontobacco users, sustain nicotine dependency via dual use, slow intentions to quit in dual users, or encourage relapse to cigarette use among former smokers [6].

Unfortunately, existing observational and epidemiological studies are largely uninformative due to severe methodological limitations. Much of the early population-level studies on adult EC use have relied on crude measures of use: (such as “once or more in the past 30 days”, or “ever use”) which do not capture frequency, intensity, or reasons for use [7–9]; such defective definitions of current EC use will include many infrequent users (mostly experimenters unlikely to use ECs regularly), thus abnormally inflating these statistics and providing misleading conclusions about the individual as well as public health impact of ECs. Notably, experimentation with ECs is now associated with an accelerated rate of decline in smoking among youths [10–12].

More realistic insights of greater public health relevance can be drawn by studies that assess, with greater precision, why, how and what smokers are using as tobacco substitutes. For example, frequency of use (daily vs non-daily) and type of EC device (advanced open tank systems vs basic cigalikes) are associated with cigarette abstinence—daily users of more efficient EC kits are most successful [13, 14]. Specifying the reason for using ECs (to quit smoking vs out of curiosity), as well as the presence of nicotine (vs its absence), are also important determinants of success [15, 16].

Some of these critical measurement issues have been addressed in a recent cross-sectional study of a representative sample of 27,801 respondents from 28 EU member states [17]. In particular, the study herein examines current daily EC use, including current daily nicotine use.

There are several important findings. Among never smokers, only minimal current daily (0.08%) and current daily nicotine-containing EC use (0.04%) is observed. By comparison, daily vaping is reported by 2.31% of current smokers and 2.18% of former smokers, the vast majority of whom reported vaping nicotine. As one would expect, daily EC use is highly prevalent among current and former smokers, but rare in never smokers. Compared to never smokers, current and former smokers are at least 50 times more likely to report daily EC use, which would suggest that vapor products are extremely unlikely to create daily nicotine dependence (just 4 in 10,000 never smokers). Thus, it does not appear that the threat of ECs attracting a new generation of nicotine addicts has materialized.

Considering that ECs are much less harmful alternatives to tobacco cigarettes, and that smoking prevalence in the 2014 Eurobarometer survey is still high at 26.4%, regular EC use of about 2% for the overall sample is disappointingly low, and it is unlikely to have the significant public health impact it could have if a harm reduction strategy was adopted by the public health community. Several factors might have contributed to such low uptake. For example, irresponsible science, careless publishing, and credulous journalism have increasingly fuelled alarmist and deeply misleading stories about potential harm of these products. These stories are now spreading fear and confusion, and have actually resulted in public perceptions shifting in the wrong direction so that ECs are now misperceived as equal to or more harmful than cigarettes, possibly resulting in some users going back to cigarettes, or not being open to even trying them. Moreover, the distortion of the scientific evidence of harms has also been misused and exaggerated thus undermining switching and slowing of speeding up the end game of eliminating smoked tobacco rather than eliminating any and all nicotine product use for adults. Promoting further access to ECs and making these products widely available may offer an opportunity to reduce or prevent some of the otherwise inevitable burden of premature death and disability caused by tobacco smoking [18].

Interactions between changes in smoking behaviour and daily EC use were also investigated. Although the cross-sectional design of the 2014 Eurobarometer survey cannot establish cause and effect, another important finding is that nearly half of all daily EC users have quit smoking completely (by vaping). Clearly, improved characterization of frequency of use as well as nicotine provision appears to play a major role when evaluating smoking cessation. A possible explanation is that regular daily EC use might have assisted many EC users to build up the necessary confidence to do something good for their health, and to stay quit or reduce cigarette consumption. The same logic may also explain the low level of relapse observed in the sample.

EC use is a complex and dynamically evolving behaviour. Its definition and detailed characterization of concurrent EC and cigarette use requires thoughtful and careful assessment. Therefore, to advance knowledge of the impact of EC use on smoking status, it will be necessary to conduct prospective studies considering relevant descriptors of vaping behaviour such as frequency of use (e.g. focusing on daily users, and not just to those who are experimenting), reasons for using ECs (e.g. to quit smoking vs out of curiosity), and product design (e.g. closed vs open systems; nicotine containing vs non-nicotine containing products; etc.). Reasons for vaping, the type of device and e-liquid, frequency of use, and the accompanying sensory and craving-control experiences will have some impact on smoking behaviors (cutting down, quitting). Only carefully conducted longitudinal studies will be able to indicate which combinations of device and human use factors are likely to lead to sustained, beneficial outcomes.

In any case the study by Farsalinos and colleagues [17] is important because by addressing some of the common methodological mistakes present in the vast majority of existing observational and epidemiological studies, it provides a more realistic estimate of current regular EC use, and of its impact on smoking habits. Despite some mixed and negative results from many early epidemiological studies with serious methodological limitations, a common theme that seems to be emerging is that if a smoker persists in seeking out and finding an EC that is satisfying to him/her (including flavors) and persists in regular use, he/she is more likely to switch or quit. By exploring diversities and similarities among different product designs, smokers are now beginning to learn that adoption rates (and consequently the extent of reduction in tobacco consumption), are intimately associated with their efficiency as smoking “sensation” products, with smoking cessation becoming a “collateral benefit” for many smokers switching to regular daily EC use [19–21].
