# Erratum to: Analysis of E-cigarette use in the 2014 Eurobarometer survey: calling out deficiencies in epidemiology methods

**DOI:** 10.1007/s11739-017-1685-x

**Published:** 2017-07-10

**Authors:** Riccardo Polosa, Pasquale Caponnetto, Ray Niaura, David Abrams

**Affiliations:** 1 Centro Prevenzione e Cura del Tabagismo, Azienda Ospedaliero, Universitaria ‘‘Policlinico-V. Emanuele’’, Università di Catania, Catania, Italy; 2 Dipartimento di Medicina Clinica e Sperimentale, Università di Catania, Azienda Ospedaliero, Universitaria ‘‘Policlinico-Vittorio Emanuele’’, Università di Catania, Catania, Italy; 3 Institute for Social Marketing, University of Stirling, Stirling, UK; 4 The Schroeder Institute for Tobacco Research and Policy Studies at Truth Initiative, Washington, DC USA; 5 Department of Health, Behavior and Society, Johns Hopkins Bloomberg School of Public Health, Baltimore, MD USA

## Erratum to: Intern Emerg Med DOI 10.1007/s11739-017-1667-z

The article Analysis of E-cigarette use in the 2014 Eurobarometer survey: calling out deficiencies in epidemiology methods, written by Riccardo Polosa, Pasquale Caponnetto, Ray Niaura, David Abrams, was originally published electronically on the publisher’s internet portal (currently SpringerLink) on May 5, 2017 without open access.

With the author(s)’ decision to opt for Open Choice the copyright of the updated version is of June 30, 2017 © The Author(s) [2017] and the article is forthwith distributed under the terms of the Creative Commons Attribution [continuing for CC BY license].

4.0 International License (http://creativecommons.org/licenses/by/4.0/), which permits use, duplication, adaptation, distribution and reproduction in any medium or format, as long as you give appropriate credit to the original author(s) and the source, provide a link to the Creative Commons license, and indicate if changes were made.

Also, the acknowledgement section should read as “Authors wish to thank Cuts Ice e-Liquid Laboratories for supporting the publication and open access costs”.

The original article has been corrected.

